# Shiitake Mushroom-Derived Vesicle-like Nanoparticles Improve Cognitive Function and Reshape Gut Microbiota and Fecal Metabolome in Aged Mice

**DOI:** 10.3390/nu17172902

**Published:** 2025-09-08

**Authors:** Xingzhi Li, Baolong Liu, Deekshika Sekar, Meghna Sur, Jay Reddy, Sathish Kumar Natarajan, Peder J. Lund, Jiujiu Yu

**Affiliations:** 1 Department of Nutrition and Health Sciences, University of Nebraska-Lincoln, Lincoln, NE 68583, USA; xxl1518@case.edu (X.L.); baolong@nwafu.edu.cn (B.L.); ds2@huskers.unl.edu (D.S.); snatarajan2@unl.edu (S.K.N.); 2 Department of Nutrition, School of Medicine, Case Western Reserve University, Cleveland, OH 44106, USA; pxl446@case.edu; 3 School of Veterinary Medicine and Biomedical Sciences, University of Nebraska-Lincoln, Lincoln, NE 68583, USA; mesur@coh.org (M.S.); nreddy2@unl.edu (J.R.)

**Keywords:** shiitake mushroom, exosomes, extracellular vesicles, cognitive function, aging, microbiota, microbiome, metabolomics, kynurenic acid

## Abstract

Background/Objectives: Population aging and its associated chronic conditions have become an unprecedented challenge in the United States and worldwide. Many aged individuals experience certain forms of cognitive decline, which increases their risk of developing a pre-dementia condition called mild cognitive impairment and even dementia. No effective pharmacological treatments are available to treat normal age-associated cognitive decline or mild cognitive impairment. Our previous study has shown the potent anti-inflammatory effects of shiitake mushroom-derived vesicle-like nanoparticles (S-VLNs) in vitro and in an acute inflammatory disease model. In this study, we aimed to investigate the potential benefits of orally administered S-VLNs in aged mice. Methods: S-VLNs were extracted from fresh shiitake mushrooms. S-VLNs in phosphate-buffered saline (PBS) or vehicle only was orally administered to 13-month-old male C57BL/6J mice weekly for 9 months. These mice were subjected to a series of physiological tests, followed by euthanasia at 22 months of age. Their fecal samples were subjected to 16S rRNA and untargeted metabolomics analyses, followed by comprehensive bioinformatics analyses. Results: The long-term oral administration of S-VLNs significantly improved the cognitive function of aged mice. Orally administered S-VLNs did not travel to the brain. Instead, they impacted the composition of the gut microbiota and reshaped the fecal metabolome. Functional predictions of the gut microbiota and fecal metabolome suggested that S-VLNs regulated tryptophan metabolism. Specifically, S-VLNs markedly decreased the tryptophan-related metabolite kynurenic acid (KYNA). The integrative analyses of omics data identified a strong correlation between 18 gut bacterial genera and 66 fecal metabolites. KYNA was found to highly correlate with five genera positively and twelve genera negatively. Conclusions: The oral intake of S-VLNs represents a new and superior dietary approach with the ability to modulate the gut microbiota and fecal metabolome and to improve cognitive function during aging.

## 1. Introduction

The number of people aged 65 or older is rapidly increasing in the United States and worldwide. A total of 58 million Americans were 65 years or older in 2022, accounting for 17% of the total population in the United States, and this number is projected to increase to 82 million by 2050 [1]. There were 771 million people aged 65 or older worldwide in 2022, and the older population is expected to reach 1.6 billion by 2050 [2]. Aging, a gradual degenerative process, is associated with many chronic conditions including cognitive decline, muscle loss, bone weakness, neurogenerative disease, metabolic disease, and cancer [3,4,5,6].

Cognitive decline is a normal process associated with aging. Some cognitive abilities, such as vocabulary, are relatively resistant to brain aging, but other abilities, such as memory, conceptual reasoning, and processing speed, decline gradually with aging [6,7]. It was estimated that approximately 40% of individuals aged 65 or older suffer from some form of memory loss [6]. Some older adults develop a pre-dementia condition called mild cognitive impairment (MCI). About one-third of individuals with MCI develop Alzheimer’s disease (AD) within five years [8].

Currently, no effective pharmacological treatments are available to manage normal age-associated cognitive decline or MCI. Lifestyle interventions, such as exercise and cognitive training, have been recommended to slow the progress of cognitive decline, but they are often challenged by low adherence and a lack of consistent effectiveness [9,10]. Growing evidence has suggested the important contribution of bidirectional communications between the gut microbiota and the brain, often termed as the “microbiota–gut–brain axis”, to cognitive decline in the normal aging process, MCI, and neurogenerative diseases [11,12]. Thus, microbiota-based therapies, including prebiotics, probiotics, and fecal microbiota transplantation (FMT), have been explored in the management of cognitive decline [11,12]. In addition, dietary interventions, such as specific nutrient supplements or controlled diets, offer another possible safe alternative to slow cognitive decline [13]. Although these new approaches have shown promising results in animal studies or small clinical trials, there is an unmet need to effectively preserve cognitive function in clinical practice.

Dietary vesicle-like nanoparticles (VLNs) or exosome-like nanoparticles are tiny membrane-enclosed nanoparticles composed of a variety of biomolecules including lipids, proteins, nucleic acids, and metabolites [14,15,16]. Accumulating evidence has demonstrated that dietary VLNs exert diverse beneficial functions after consumption. For instance, tea flower VLNs potently inhibited tumor growth in a mouse breast tumor model [17]. The oral administration of honey VLNs prevented the liver of aged mice from developing metabolic syndrome-associated steatohepatitis [18]. Further, milk VLNs promoted spatial learning and memory in mice [19]. In our previous study [20], we successfully extracted VLNs from shiitake mushrooms (S-VLNs). These S-VLNs showed potent anti-inflammatory functions in cell culture. A single intraperitoneal injection of S-VLNs protected mice from D-galactosamine and lipopolysaccharide-induced acute hepatic inflammation and liver injury.

Although we demonstrated the potent anti-inflammatory actions of S-VLNs in vitro and in vivo, it was unclear whether the regular oral intake of S-VLNs could have any long-term beneficial effects. The purpose of this study was to address this question by orally administering S-VLNs weekly to aged mice. Aging is a natural degenerative process with gradual functional decline in multiple tissues. Therefore, aged mice provide a unique opportunity to assess the potential beneficial functions of S-VLNs in different tissues. Interestingly, we found that S-VLNs improved cognitive function and reshaped the gut microbiota and fecal metabolome in aged mice. Our findings are highly significant considering the lack of effective strategies to combat cognitive decline in clinical settings. The oral intake of S-VLNs represents a potential cost-effective and user-friendly approach to preserve cognitive function.

## 2. Materials and Methods

### 2.1. Extraction and Characterization of S-VLNs

Fresh shiitake mushrooms (*Lentinula edodes*) were obtained from local grocery stores and subjected to VLN extraction as previously described [20]. Briefly, the whole mushrooms, including both their cap and stem, were washed and minced. Three grams of minced mushrooms were homogenized twice (15 s each time) in 30 mL of cold phosphate-buffered saline (PBS, VWR, Radnor, PA, USA) using a kitchen blender. After filtering through a mesh, the mushroom juice was subjected to sequential centrifugations at 4 °C: 500× *g* for 10 min and 2000*× g* for 20 min using an Allegra X-30R centrifuge (Beckman Coulter, Brea, CA, USA), and 10,000× *g* for 30 min and 100,000× *g* for 2 h using an SW-32TI rotor in an Optima XE-90 ultracentrifuge (Beckman Coulter). The VLN pellet was rinsed with cold PBS, resuspended in cold PBS, and filtered through a 200 nm Acrodisc filter (Pall Laboratory, Port Washington, NY, USA) to sterilize the solution.

The yield and size of S-VLNs were measured using a NanoFCM NanoAnalyzer (Xiamen, Fujian, China) per the manufacturer’s instructions. For zeta potential measurement, S-VLNs in PBS were diluted using double-distilled water (1:1000, *v*/*v*) to a final concentration of 1 × 10^9^/mL (pH 7.4) and measured using a Litesizer 500 Particle Analyzer (Anton Paar, Vernon Hills, IL, USA). Ultrastructure transmission electron microscopy (TEM) analysis of S-VLNs was conducted as previously reported [21]. The images were taken using a Hitachi H7500 TEM with a bottom-mount AMT digital camera.

For biomolecule analysis, S-VLN proteins were extracted using a lysis buffer containing 150 mM NaCl, 50 mM Tris-HCl (pH 7.5), and 1% NP-40. The protein samples were loaded and separated with a NuPAGE 4–12% Bis-Tris protein gel (ThermoFisher, Waltham, MA, USA) using the NuPAGE MOPS Running Buffer (ThermoFisher). The protein bands were visualized using Coomassie blue staining. S-VLN lipids were extracted with a mixture of chloroform/methanol (2:1, Sigma, St. Louis, MO, USA). The organic phase containing the total lipids was collected, dried, and redissolved in chloroform. The lipid samples were separated on a silica gel thin-layer chromatography (TLC) plate (EMD Millipore, Burlington, MA, USA) using a mixture of chloroform/methanol/water (65:25:4) and visualized with 10% CuSO_4_ in 8% phosphoric acid (Sigma). S-VLN RNAs were extracted using a miRNeasy Mini Kit (Qiagen, Germantown, MD, USA) and separated on a 2.5% agarose gel.

### 2.2. Mice

20 four-week-old male C57BL/6J mice from Jackson Laboratory (Bar Harbor, ME, USA) were housed under specific pathogen-free conditions and fed with a Teklad chow diet (Inotiv, 2016, West Lafayette, IN, USA). Plastic bones and huts were kept in each cage as enrichments. Animal studies were approved by the Institutional Animal Care and Use Committee (IACUC) of the University of Nebraska–Lincoln (Project ID 2054, approved on 1 March 2021). Upon reaching 13 months of age, these 20 age-matched mice were randomly assigned to two groups as determined by coin toss (10 mice per group). The sample size was determined based on our previous study about the effects of honey VLNs in aged mice [18]. The control group (3 cages, 2–5 mice/cage) received PBS and the treatment group (3 cages, 2–5 mice/cage) received S-VLNs in PBS at a dose of 1 × 10^10^/g of body weight via oral gavage weekly. These 6 cages were randomly placed on the same row on multi-level racks. Each mouse was considered as an experimental unit, and its body weight and general health were monitored weekly. Aging, as a natural process, was not considered to cause any suffering or distress to the mice. All the mice were planned to be euthanized when they reached 22 months of age. However, if any mouse kept losing its body weight in two sequential weeks, or develop tumors, or other health issues, they would be reported, followed by removal from the study and humanly euthanasia. During the study, two mice in the control group and 3 mice in the treatment group died possibly due to aging.

After 6 months of S-VLN treatment, the bone density of the thigh bones of each mouse was assessed using an UltraFocus DXA in vivo imaging system (BioVision, Milpitas, CA, USA). After two weeks, metabolic cage analysis was conducted with all the mice using a Phenomaster/Labmaster caging system (TSE Systems, Chesterfield, MO, USA) for 48 h. Mouse adaptation to the metabolic cages occurred during the first 24 h, and the metabolic data collection occurred during the next 24 h. During the metabolic data collection period, food and water intake, oxygen consumption, CO_2_ production, respiratory exchange ratio (RER), heat production, and movements of each mouse were recorded every 30 min.

After 7 months of S-VLN treatment, the mice were subjected to an intraperitoneal glucose tolerance test (ipGTT). The mice were fasted for 6 h before they received an intraperitoneal injection of glucose at a dose of 1.5 g/kg. Blood glucose levels were measured every 15 min in the first 60 min and then every 30 min in the next 90 min using a Bayer Contour Next blood glucose monitoring system (Parsippany, NJ, USA). After one week, the mice were subjected to a run endurance test to assess their physical fitness. Briefly, the mice were trained on a flat treadmill (Panlab, Holliston, MA, USA) starting at a speed of 5 m/min, with an incremental increase of 0.5 m/min every 10 min, up to 6.5 m/min, for 40 min for four consecutive days. On day 5, the mice started to run at 7 m/min with an increase of 0.9 m/min every 10 min until they were considered exhausted (i.e., resting for more than 30 s at the rear of the treadmill in spite of air puff stimulation). Two weeks later, the cognitive function of the mice was assessed using the Barnes maze test. The maze table with surrounding visual cues was placed in a well-lit room. The visual cues remained unaltered during the entire experiment. Mice were trained to locate an escape tunnel underneath a specific hole of the maze table for 6 consecutive days. On day 7, each mouse was placed in the middle of the maze table and the time needed by them to find the escape tunnel was recorded. Between each mouse trial, the maze table and escape tunnel were thoroughly cleaned using 70% ethanol to avoid any olfactory cues. The mice were returned to their original cages and rested for one week. On day 14, each mouse was tested on the maze table again, and the time needed by them to find the escape tunnel was recorded.

For each physiological test, all live mice were included and the experimental time was predetermined to minimize possible pain, suffering, or distress. For the endurance run test, the painless air puff stimulation was used. When the mice rested in spite of the air puff stimulation, they were considered exhausted and removed from the treadmill. The test order was one mouse from the control group followed by one mouse from the treatment group to minimize the effects of treatment order. The animals were not blinded during the physiological tests and data analysis.

When the mice reached 22 months of age, the remaining 8 mice in the control group and 7 mice in S-VLN-treated group were euthanized using compressed carbon dioxide gas in cylinders. A variety of tissues and their fecal samples were snap-frozen in liquid nitrogen. Alternatively, the major metabolic tissues, including the liver, kidneys, heart, and gastrocnemius muscle, were fixed in 10% formalin (VWR) for further analyses.

For all the physiological tests, the data were presented as the mean ± SEM. A two-tailed *t*-test in the software Excel was conducted for comparisons. *p* < 0.05 was considered statistically significant and indicated by *; *p* < 0.01 was indicated by **.

### 2.3. Tissue Hematoxylin and Eosin (H&E) Staining

A small piece of the liver, heart, kidney, and gastrocnemius muscle was fixed in 10% formalin solution (VWR, Radnor, PA, USA) overnight. Afterward, the tissues were transferred to cold PBS and delivered to the Veterinary Diagnostic Laboratory at the University of Nebraska–Lincoln. The tissues were embedded in paraffin, cut into 8 μm-thick sections, and subjected to routine H&E staining. The images of H&E-stained sections were taken using an EVOS M5000 imaging system (ThermoFisher).

### 2.4. Biodistrubtion of S-VLNs In Vivo

The S-VLN biodistribution study was conducted as previously described [22]. Animal studies were approved by the IACUC of the Case Western Reserve University (Project ID 2023-0059, approved on 10 April 2024). Briefly, S-VLNs were covalently labeled with a fluorescent dye in near-infrared ranges using the ExoGlow-Vivo EV Labeling Kit (System Biosciences, EXOGV900A-1, Palo Alto, CA, USA) per manufacturer’s instructions. Four-month-old male C57BL/6J mice were orally administered with PBS or fluorescent-labeled S-VLNs in PBS at a dose of 79,300 fluorescence intensity/g. Six hours later, the mice were euthanized to collect their gastrointestinal tract, brain, heart, kidneys, lungs, spleen, and liver. The fluorescent signals of organs were measured using an Odyssey Clx imaging platform (LI-COR Biosciences, Lincoln, NE, USA).

### 2.5. 16S Ribosomal RNA (rRNA) Analyses of Mouse Fecal Samples

The fecal samples of control and S-VLN-treated aged mice were subjected to genomic DNA extraction using the QIAamp DNA Stool Mini Kit (Qiagen). The hypervariable regions V3-V4 of the 16S rRNA gene were amplified using PCR. After purification and quantification of PCR products, the DNA library was constructed. Amplicons were performed on a paired-end Illumina MiSeq platform to generate 300 bp paired-end raw reads, which were assigned to a sample using unique barcodes. The barcodes and primer sequences were then truncated. The paired-end reads were merged using FLASH (v1.2.11), followed by quality filtering using Fastp to obtain the high-quality clean reads. Chao1 and Shannon metrics were calculated to assess the alpha diversity. The Non-Metric Multi-Dimensional Scaling (NMDS) analysis based on Bray–Curtis or weighted UniFrac was used to evaluate the beta diversity. The taxonomic classification was conducted through QIIME II using the Silva rRNA database. Phylogenetic Investigation of Communities by Reconstruction of Unobserved States (PICRUSt) was conducted to predict the potential functional changes in the gut microbiota.

### 2.6. Untargeted Metabolomics Analyses of Mouse Fecal Samples

Two 5 mm metal beads and 80% methanol at 8 μL/mg were added to each fecal sample of control and S-VLN-treated aged mice. All samples were ground for 3 min at 65 Hz, followed by sonication for 30 min at 4 °C. Afterward, the samples were kept at −20 °C for 1 h, vortexed for 30 s, and centrifuged at 12,000 rpm for 10 min at 4 °C. The supernatant was mixed with 0.14 mg/mL DL-O-Chlorophenylalanine in a ratio of 40:1 and filtered through a 200 nm filter. Two microliters of the filtered samples were injected into a Vanquish Flex Ultra-Performance Liquid Chromatography (UPLC) combined with Q Exactive plus mass spectrometer (MS, ThermoFisher) operating in the electrospray ionization positive (ESI^+^) or negative (ESI^-^) mode separately. An ACQUITY UPLC HSS T3 column (100 × 2.1 mm × 1.8 μm) was used. The mobile phase was composed of solvent A (0.05% formic acid) and solvent B (acetonitrile) with a gradient elution (0–1 min, 5% B; 1–12 min, 5–95% B; 12–13.5 min, 95% B; 13.5–13.6 min, 95–5% B; 13.6–16 min, 5% B). The flow rate of the mobile phase was 0.3 mL/min. Full scan (MS1, m/z 70–1050, resolution: 70,000) and data dependent-MS2 (dd-MS2, TopN = 10, resolution: 17,500) mass scan acquisition modes were used. In dd-MS2, the collision energy was set at 15, 30, and 45 eV. The parameters for the ESI^+^ mode were as follows: Heater Temperature: 300 °C; Sheath Gas Flow Rate, 45 arb; Aux Gas Flow Rate: 15 arb; Sweep Gas Flow Rate: 1 arb; Spray Voltage: 3.0 kV; Capillary Temperature: 350 °C; and S-Lens RF Level: 30%. The parameters for the ESI^-^ mode were as follows: Heater Temperature: 300 °C; Sheath Gas Flow Rate: 45 arb; Aux Gas Flow Rate, 15arb; Sweep Gas Flow Rate: 1 arb; Spray Voltage: 3.2 kV; Capillary Temperature: 350 °C; and S-Lens RF Level: 60%.

The raw data were acquired, and the metabolites were annotated using the Compound Discoverer (3.0, ThermoFisher) based on the m/z value and the retention time of precursor ions and fragment ions. Ions from both ESI^-^ and ESI^+^ were merged and imported into the SIMCA-P program (v.14.1) for multivariate analysis. Orthogonal partial least squares discriminant analysis (OPLS-DA) was used to compare the metabolic differences between the PBS control group and the S-VLN-treated group. Metabolites with variable importance in projection (VIP) > 1.5, fold change > 2, and *p* < 0.05 were selected for hierarchical clustering analysis (HCA) using the complete linkage algorithm of the program Cluster 3.0 [23] and the results were visualized using Pheatmap 1.0.12. These metabolites were also used to conduct metabolic pathway enrichment analysis under the limiting condition of *p* < 0.05 in the MetaboAnalyst 6.0.

### 2.7. Integrative Analyses of 16S rRNA Sequencing Data and Untargeted Metabolomics Data

The data from the fecal metabolome and 16S rRNA sequencing were further subjected to integrative analyses. First, Pearson correlation analysis was conducted to assess the linear correlation between fecal metabolites and microbial composition. Microbe–metabolite relationship pairs satisfying the absolute value of Spearman rank correlation coefficient |r| ≥ 0.7 and *p* < 0.05 were selected for the correlation chord diagram. These pairs were also subjected to HCA as described in Section 2.6. The top 10 metabolites and microbial genera with the highest correlation coefficients and *p* < 0.05 were selected as variables to draw the redundancy analysis (RDA) diagram.

## 3. Results

### 3.1. Characterization of S-VLNs

**Figure 1:**
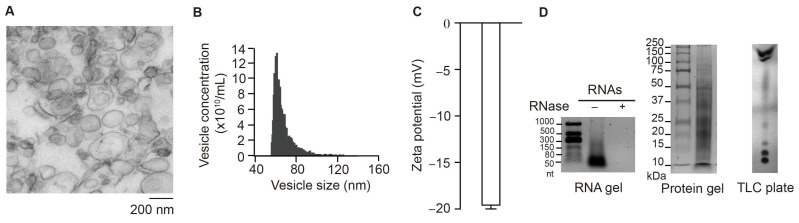
Characterization of S-VLNs. (**A**) Representative images of ultrastructure TEM of S-VLNs. (**B**) NanoFCM NanoAnalyzer showed the yield and size of S-VLNs. (**C**) Zeta potential of S-VLNs. Data are presented as mean ± SEM (*n* = 3). (**D**) Biomolecule composition of S-VLNs. RNAs, proteins, or lipids were extracted from S-VLNs. RNAs were incubated without (−) or with RNase (+) for 30 min at 37 °C and then loaded and run on an agarose gel. nt: nucleotides. S-VLN proteins were run on a Bis-Tris protein gel. S-VLN lipids were separated on a TLC silica gel plate.

S-VLNs were extracted from fresh shiitake mushrooms using the ultracentrifugation method [20]. The nanoparticles were subjected to ultrastructure TEM, which showed that S-VLNs had a membrane-enclosed vesicle or exosome-like structure (Figure 1A). NanoFCM NanoAnalyzer showed that the peak sizes of S-VLNs were around 60 nm in diameter (Figure 1B). The zeta potential of S-VLNs was approximately −20 mV (Figure 1C). Biomolecule analysis showed that S-VLNs contained small-sized RNAs and a variety of proteins and lipids (Figure 1D), which were consistent with our previous findings [20].

### 3.2. Oral Administration of S-VLNs Improved the Cognitive Function of Aged Mice

**Figure 2:**
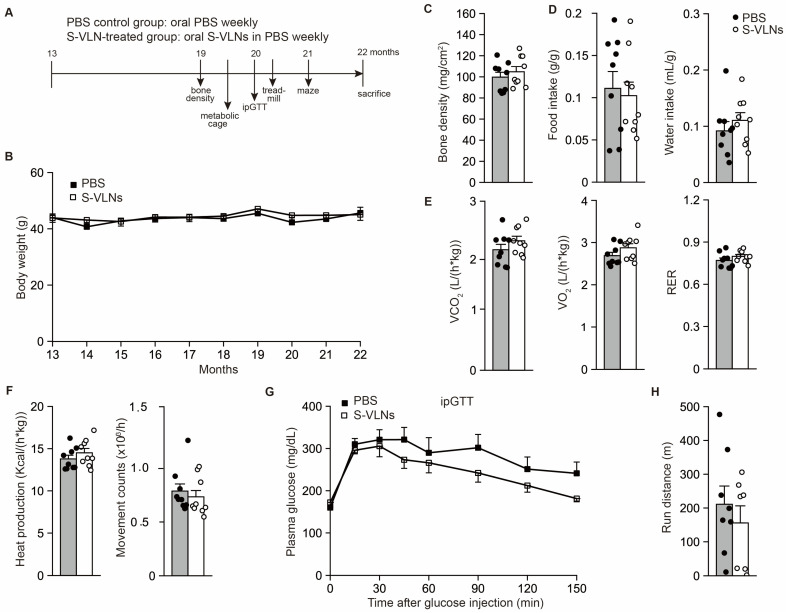
S-VLNs had no significant impact on the results of a number of physiological and metabolic tests. (**A**) Schematic diagram of the animal studies. Thirteen-month-old male C57BL/6J mice were orally administered with the solvent PBS (PBS control group) or 1 × 10^10^/g S-VLNs in PBS (S-VLN-treated group) weekly (*n* = 10/group). The mice underwent several physiological and metabolic tests and were euthanized when they reached 22 months of age. Maze: the Barnes maze test. (**B**) Body weight of the mice between 13 and 22 months of age. *n* = 7–10/group. (**C**) Bone density of the mice. The bone density of the thigh bones was measured after 6 months of S-VLN treatment. *n* = 9/group. (**D**–**F**) Food intake, water intake, O_2_ consumption rate, CO_2_ production rate, RER, heat production, and movement counts of aged mice. Individual mice were kept in metabolic cages for 48 h. The first 24 h was the adaptation period, and the last 24 h was the data collection period. *n* = 9/group. (**G**) The ipGTT results of aged mice. The test was conducted after 7 months of S-VLN treatment. The mice were fasted for 6 h, followed by the intraperitoneal injection of 1.5 g/kg glucose. The blood glucose levels were measured over the next 150 min. *n* = 8/group. (**H**) Run distance of aged mice in an endurance test. *n* = 7–8/group. In the bar graphs, each dot represents one mouse. Data are presented as mean ± SEM.

Thirteen-month-old male C57BL/6J mice were randomly assigned to two groups: the control group received PBS and the treatment group received S-VLNs in PBS through oral gavage weekly for 9 months (Figure 2A). During the entire experiment, the body weights of the mice in the control and treatment groups were monitored and found to be comparable (Figure 2B). After 6 months of S-VLN treatment, the thigh bones of mice were assessed for their bone density. There were no remarkable differences in the thigh bone density between the two groups (Figure 2C). Two weeks later, the mice were transferred to metabolic cages to measure their metabolic parameters. The food and water intake, oxygen consumption, CO_2_ production, RER, heat production, and movements of the mice in the control and S-VLN-treated groups were comparable (Figure 2D–F), indicating that the long-term oral administration of S-VLNs was safe and had no adverse impact on overall mouse health. Meanwhile, such neutral results suggested that S-VLNs had no beneficial effects on these metabolic parameters.

**Figure 3:**
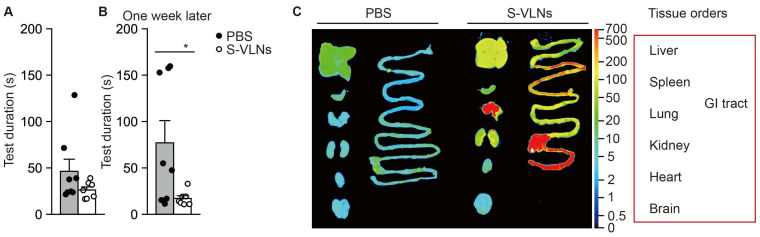
S-VLNs improved cognitive function of aged mice but did not travel to the brain. (**A**,**B**) The Barnes maze test of aged mice. The same mice from Figure 2 were further subjected to the Barnes maze test. *n* = 7–8/group. After 8 months of S-VLN treatment, the mice were trained for six days to find the escape tunnel on the Barnes maze table. On day 7, each mouse was tested and the time needed by them to find the escape tunnel was recorded (**A**). The mice were returned to their cages and rested for one week. On day 14, each mouse was tested again on the Barnes maze table to record the time they needed to find the escape tunnel (one week later, (**B**)). Each dot represents one mouse. Data are presented as mean ± SEM. * *p* < 0.05 compared with the PBS control group (bar with black dots). (**C**) Orally administered S-VLNs accumulated in the intestinal tract but not the brain. S-VLNs were covalently linked to a fluorescent dye in the near-infrared ranges. The resulting labeled S-VLNs were orally given to C57BL/6J mice. Six hours later, the mice were euthanized, and their gastrointestinal (GI) tract, brain, heart, kidneys, lungs, spleen, and liver were collected. The fluorescent images of the organs were taken using an Odyssey Clx imaging system.

After 7 months of S-VLN treatment, an ipGTT was conducted with these mice. S-VLN-treated mice tended to have improved glucose homeostasis, but the difference did not reach statistical significance (Figure 2G). One week later, the mice were subjected to an endurance run test. The average run distances were similar between the two groups (Figure 2H). Two weeks later, the mice were assessed using the Barnes maze test. After one week of training, the mice in both groups took a similar amount of time to locate the escape tunnel underneath a specific hole of the maze table (Figure 3A). Notably, after one week of rest, the mice in the S-VLN-treated group spent significantly less time finding the escape tunnel (Figure 3B), indicating that the oral administration of S-VLNs improved memory retention in these aged mice.

When the mice reached 22 months of age, they were euthanized. To assess whether S-VLNs had any toxic effects in animals, the major metabolic tissues, including the liver, heart, kidneys, and gastrocnemius muscle, were collected for microscopic examination. The tissue structure and cellular morphology of the liver, heart, kidneys, and gastrocnemius muscle were comparable between the control and S-VLN-treated groups (Figure S1). These histological results confirmed that the long-term (9 months) oral administration of S-VLNs was well tolerated and caused no morphological changes or pathological lesions in major metabolic tissues.

### 3.3. Orally Administered S-VLNs Did Not Reach the Brain

Next, we assessed S-VLN biodistribution to understand how S-VLNs exerted their memory-protective function. The fluorescent-labeled S-VLNs in PBS or PBS alone was orally administered to C57BL/6J mice. After 6 h, the mice were euthanized to collect their intestinal tract, brain, heart, kidneys, lungs, spleen, and liver. Consistent with the results from other dietary VLNs [18,22], the fluorescent signals of S-VLNs were readily detected in the gastrointestinal tract (Figure 3C). In addition, the fluorescent signals of S-VLNs were found in the kidneys, lungs, and liver but not in the brain, heart, and spleen (Figure 3C). Together, the data suggested that S-VLNs did not travel to the brain to directly exert their functions, and the memory-protective properties of S-VLNs may be achieved through indirect modulation.

### 3.4. S-VLN Intake Significantly Reshaped Gut Microbiota

As S-VLNs accumulated in the intestinal tract and did not travel to the brain, we hypothesized that S-VLNs may exert their functions through the microbiota–gut–brain axis. To test our hypothesis, the fecal samples of the control and S-VLN-treated aged mice were subjected to 16S rRNA analysis and untargeted metabolomics analyses, followed by comprehensive bioinformatics analyses.

**Figure 4:**
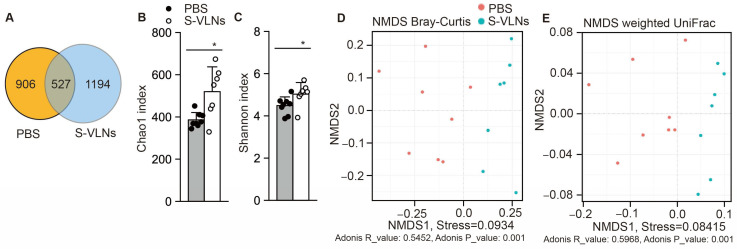
S-VLN treatment increased complexity and diversity of gut microbiota. The fecal samples of the same mice from Figure 2 were subjected to 16S rRNA analysis, followed by the alpha and beta diversity assessment. *n* = 7–8 mice/group. (**A**) Venn diagram showed that 527 ASVs were shared by both groups, and 906 and 1194 ASVs were uniquely identified in the PBS control and S-VLN treatment groups, respectively. (**B**–**E**) The alpha diversity and beta diversity were conducted. (**B**) Chao1 index. (**C**) Shannon index. (**D**) NMDS analysis based on Bray–Curtis. (**E**) NMDS analysis based on weighted UniFrac. In the bar graphs, each dot represents one mouse. Data are presented as mean ± SEM. * *p* < 0.05 compared with the PBS control group (bar with black dots).

16S rRNA analysis identified 1433 and 1721 amplicon sequence variants (ASVs) in the control and S-VLN-treated groups, respectively. Among them, 527 ASVs were shared between two groups; a total of 906 and 1194 unique ASVs were present in the control and S-VLN-treated groups, respectively (Figure 4A). The alpha diversity was assessed using Chao1 and Shannon metrics, which suggested that both richness and evenness of microbial population were significantly increased by the S-VLN treatment (Figure 4B,C). The beta diversity was evaluated using the NMDS analysis based on either Bray–Curtis or weighted UniFrac. Both of these analyses showed that the mice in the same group clustered together, and the treatment group was separated from the control group (Figure 4D,E), suggesting that the microbial composition differed between the two groups.

**Figure 5:**
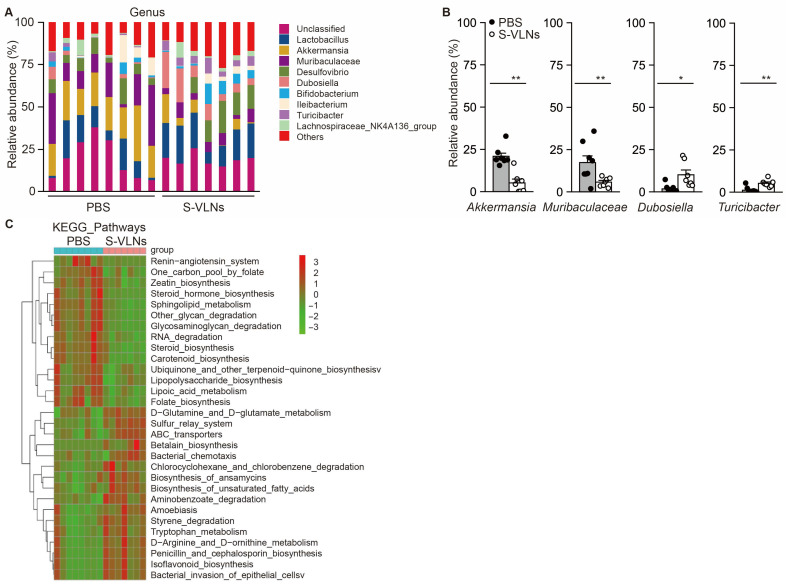
The S-VLN treatment significantly changed the composition and potential functions of the gut microbiota. The fecal samples of the same mice from Figure 2 were subjected to 16S rRNA analysis, followed by taxonomic composition and PICRUSt analyses. *n* = 7–8 mice/group. (**A**) The histogram of taxonomy distribution at the genus classification level. Each color represents a taxonomy, and the length of the colored blocks indicated the relative abundance of the taxonomy. Only the top nine most abundant genera are displayed in the histogram. *Muribaculaceae* is used to represent a strain from the *Muribaculaceae* family whose genus name has not yet been determined but its sequences differed from the other known genera in this family. (**B**) S-VLNs significantly reduced the abundance of *Akkermansia* and the genus with an undetermined name from the family *Muribaculaceae* but increased the abundance of *Dubosiella* and *Turicibacter* in the top nine most abundant genera displayed in (**A**). (**C**) PICRUSt predicted the functional changes in the gut microbiota by S-VLNs. In the bar graphs, each dot represents one mouse. Data are presented as mean ± SEM. * *p* < 0.05 and ** *p* < 0.01 compared with the PBS control group (bar with black dots).

Next, the taxonomic composition of the gut microbiota was analyzed. The S-VLN treatment significantly changed the level of six phyla among the top nine most abundant phyla (Figure S2A). Specifically, S-VLNs markedly decreased Bacteroidota and Verrucomicrobiota but increased Firmicutes, Actinobacteriota, Patescibacteria, and Cyanobacteria (Figure S2B). Among the top nine most abundant genera, S-VLNs dramatically decreased the levels of *Akkermansia* and a genus with an undetermined name from the family *Muribaculaceae* but increased the abundance of *Dubosiella* and *Turicibacter* (Figure 5A,B). In total, the levels of 33 genera were significantly changed by S-VLNs (Table S1). The levels of 28 genera, such as *Faecalibaculum*, *Roseburia*, *Candidatus*, *Dubosiella*, and *Eubacterium*, were enhanced, and the abundance of five genera, including *Harryflintia*, *Odoribacter*, *Muribaculaceae*, *Akkermansia*, and *Muribaculum*, was reduced by S-VLNs.

PICRUSt was conducted to predict the potential functional changes in gut microbiota. Among the top 30 Kyoto Encyclopedia of Genes and Genomes (KEGG) pathways significantly influenced by S-VLNs, 14 pathways, such as lipopolysaccharide biosynthesis and steroid biosynthesis, were downregulated, whereas 16 pathways, including tryptophan metabolism and biosynthesis of unsaturated fatty acids, were upregulated (Figure 5C).

### 3.5. S-VLN Intake Had a Significant Impact on the Fecal Metabolome

**Figure 6:**
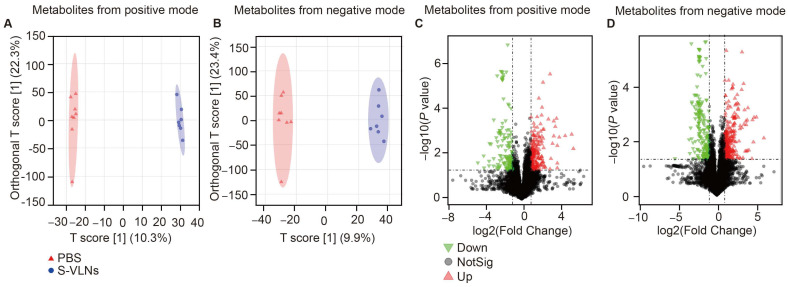
The S-VLN treatment remodeled the landscape of fecal metabolites. The fecal samples of the aged mice from Figure 2 were subjected to untargeted metabolomics analysis. *n* = 7–8 mice/group. (**A**,**B**) The OPLS-DA score plots of metabolites from the ESI^+^ mode (**A**) and ESI^-^ mode (**B**). (**C**,**D**) Volcano plots of metabolites from the positive mode (**C**) or negative mode (**D**).

Untargeted metabolomics analyses of the fecal samples found that 665 (out of 9090) metabolites from the ESI^+^ mode and 897 (out of 11538) from the ESI^-^ mode were annotated using the Compound Discover (Table S2). The OPLS-DA score plots of fecal metabolites from either the positive or negative mode showed a clear separation between the control and S-VLN-treated groups (Figure 6A,B). Volcano plots showed that many metabolites were upregulated or downregulated by the S-VLN treatment (Figure 6C,D).

**Figure 7:**
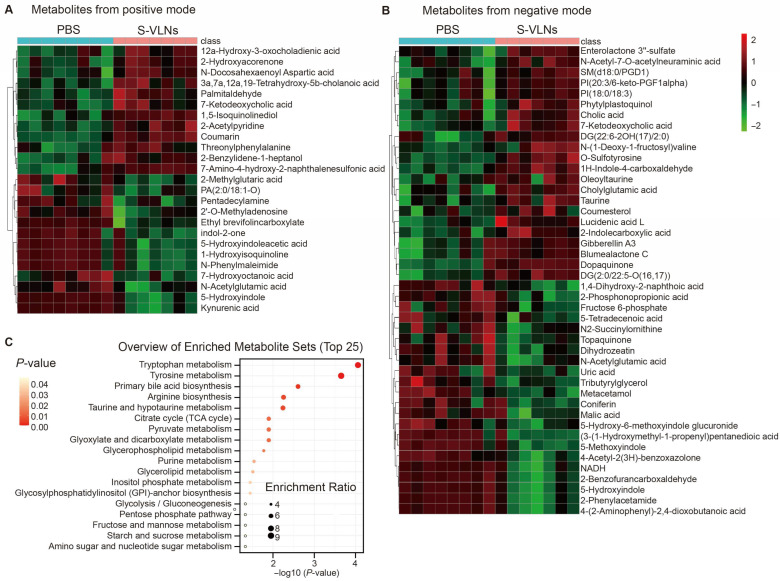
S-VLNs significantly changed the levels of 25 positively and 44 negatively charged metabolites. The fecal samples of the same mice from Figure 2 were subjected to untargeted metabolomics analysis, followed by HCA and pathway enrichment analyses. *n* = 7–8 mice/group. (**A**,**B**) The annotated metabolites with VIP > 1.5, fold change > 2, and *p* < 0.05 were considered as significantly changed metabolites by S-VLNs and subjected to HCA. Color intensity correlates with the degree of increase (red) and decrease (green) relative to the mean metabolite ratio. (**A**) Metabolites from the positive mode. (**B**) Metabolites from the negative mode. (**C**) Pathway enrichment analysis using MetaboAnalyst showed that fecal metabolites significantly regulated by S-VLNs were mainly involved in 18 metabolic pathways.

The annotated metabolites with VIP value > 1.5, fold change > 2, and *p* value < 0.05 were considered as significantly changed metabolites. HCA of these metabolites showed that the levels of 12 metabolites from the ESI^+^ mode and 22 from the ESI^-^ mode were significantly increased by S-VLNs, whereas the abundance of 13 metabolites from the positive mode and 22 from the negative mode was markedly suppressed by S-VLNs (Figure 7A,B). Pathway enrichment analysis in the MetaboAnalyst showed 18 metabolic pathways markedly regulated by S-VLNs (Figure 7C). Among them, the top five metabolic pathways were tryptophan metabolism, tyrosine metabolism, primary bile acid biosynthesis, arginine biosynthesis, and taurine and hypotaurine metabolism. Notably, tryptophan metabolism was also predicted to be regulated by S-VLNs using the PICRUSt tool (Figure 5C). Scrutinization of fecal metabolites (Figure 7A,B) identified that two kynurenine pathway-related metabolites, 4-(2-Aminophenyl)-2,4-dioxobutanoic acid and kynurenic acid (KYNA), were markedly decreased by S-VLNs. 90% of circulating tryptophan is metabolized through the kynurenine pathway [24,25]. KYNA is one of the end products of the kynurenine pathway, whereas 4-(2-Aminophenyl)-2,4-dioxobutanoic acid is an intermediate metabolite of this pathway [26].

### 3.6. Integrative Analyses of Gut Microbiota and Fecal Metabolome

**Figure 8:**
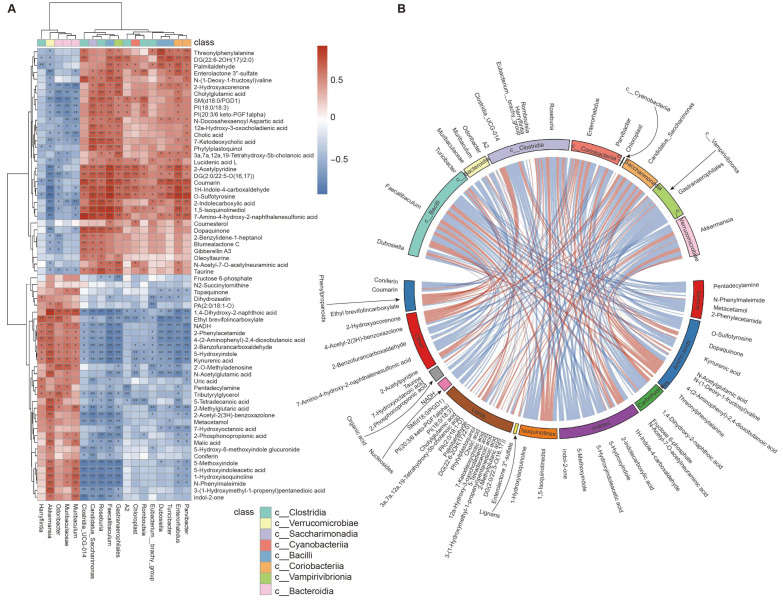
Correlation analyses of the gut microbiota and fecal metabolome. The fecal samples of the aged mice from Figure 2 were subjected to 16S rRNA and untargeted metabolomics analyses, which were further subjected to integrative analyses. *n* = 7–8 mice/group. (**A**) The HCA heatmap showed 66 fecal metabolites highly correlated with 18 bacterial genera. Red indicates a positive correlation, and blue indicates a negative correlation. * *p* < 0.05, ** *p* < 0.01. (**B**) The Spearman correlation chord diagram depicted the strength and direction of the correlation between the bacterial genera and fecal metabolites. The node data were arranged radially along the circumferences and modes were linked using weighted (width) arcs. The arc color indicates a positive (red) or a negative (blue) correlation and its width indicates the strength of the correlation. Only highly correlated pairs (|r| ≥ 0.7 and *p* < 0.05) were included and displayed in the chord diagram.

Comprehensive integrative analyses were conducted to evaluate the relationship between the gut microbiota and fecal metabolome. Spearman’s rank correlation coefficient r was calculated for each pair of the relative abundance of bacterial genus and metabolite. The value r > 0 indicated positive correlation and r < 0 meant negative correlation. The absolute value |r| ≥ 0.7 suggested a high correlation between the two variables [27]. A total of 66 fecal metabolites were frequently found to highly correlate with 18 bacterial genera (Table S3). The correlated pairs were clustered and visualized using a heatmap (Figure 8A). Noteworthily, KYNA was found to highly correlate with five genera positively and twelve genera negatively (Figure 8A). The chord diagram further displayed the strength and direction (positive or negative) of the connections between the bacterial genera and fecal metabolites (Figure 8B). The top 10 metabolites and the top 10 microbial genera with the highest Spearman’s rank correlation coefficients and *p* < 0.05 were selected for RDA. The RDA diagram visualized the strength and direction of correlations of genera and metabolites (Figure S3). KYNA was one of the top 10 metabolites and was shown to positively correlate with *Odoribucter* and *Akkermansis* but negatively correlate with several genera such as *Dubosiella*, *Enterorhabdus*, and *Parvibacter.*

## 4. Discussion

In summary, the weekly oral administration of S-VLNs for nine months improved the cognitive function in 22-month-old mice. Orally administered S-VLNs were readily detected in the intestinal tract but not in the brain. The detailed omics analysis of fecal samples demonstrated that the oral administration of S-VLNs in aged mice remarkably remodeled the gut microbiota composition. The landscape of fecal metabolite profiles was significantly modified by S-VLNs. Integrative analysis revealed strong correlations between certain bacterial genera and fecal metabolites.

The consumption of dietary VLNs has beneficial effects in a wide range of diseases, such as colitis, cancer, metabolic syndrome-associated steatohepatitis, and alcohol-associated liver disease [14,15]. However, only limited kinds of dietary VLNs showed beneficial functions in the brain. Orally administered oat VLNs directly traveled to the brain and improved memory in alcohol-fed mice [28]. Milk EVs crossed the blood–brain barrier, accumulated in the brain, and contributed to spatial learning and memory in mice [19]. Intravenous injection of ginseng VLNs led to their rapid accumulation in the brain of mice and inhibited glioma progression [29]. Intravenously administered Panax notoginseng VLNs were found in the brain of rats and attenuated cerebral ischemia/reperfusion injury [30]. In this study, the oral intake of shiitake mushroom VLNs improved memory in aged mice (Figure 3). Unlike VLNs from oat, milk, and ginseng, S-VLNs did not directly travel to the brain to exert their functions (Figure 3). Instead, they markedly reshaped the gut microbiota composition, as well as fecal metabolome (Figure 4, Figure 5, Figure 6 and Figure 7). Therefore, S-VLNs may achieve their memory-retention actions in aged mice through the microbiota–gut–brain axis.

It has been well recognized that gut microbiota’s composition and functions change significantly with aging and/or cognitive function. In humans, the abundance of phylum Actinobacteria markedly decreased with aging and phylum Bacteroidetes increased in humans beyond 70 years of age [31]. In AD patients, a decrease in Firmicutes and Actinobacteria and an increase in Bacteroidetes were documented [32]. Firmicutes was positively linked to memory, while Bacteroidetes was negatively correlated with memory [33]. Interestingly, we found that S-VLN intake increased the abundance of Firmicutes and Actinobacteria but decreased the abundance of Bacteroidetes (Figure S2), resulting in a microbiota composition that favors memory retention.

At the genus level, S-VLNs significantly decreased the abundance of *Akkermansia* and a genus with an undetermined name from the family *Muribaculaceae* but increased the abundance of *Dubosiella* and *Turicibacter* in the top nine most abundant genera (Figure 5). The genus *Akkermansia* has been found to improve cognitive dysfunction in AD rodent models [34,35]. A recent study showed that administration of two *Akkermansia* strains improved spatial working memory in aged mice [36]. Such evidence suggested that an S-VLN-mediated decrease in *Akkermansia* may not represent a favorable shift for cognitive function. It would be interesting to study how S-VLNs affect the metabolic traits or functions of *Akkermansia* in the future. The genus *Dubosiella* was greatly reduced in AD mouse models [37,38] and the level of genus *Turicibacter* decreased and correlated with cognitive impairment in both patients with AD [39] and patients with major depressive disorder [40]. Therefore, an S-VLN-mediated increase in these two genera was likely to be beneficial for cognitive function. For less abundant genera, S-VLNs decreased the abundance of *Muribaculum*, and increased the level of *Faecalibaculum*, *Roseburia*, *Candidatus*, and *Eubacterium* (Table S1), all of which have been implicated in aging and cognitive function. For example, the genus *Muribaculum* was shown to increase during AD progression in mice and was negatively associated with cognitive function [41]. The abundance of *Faecalibaculum* was positively associated with cognitive function [42,43]. A decrease in the genus *Roseburia* was accompanied by cognitive decline [42], and a treatment that curbed diabetes-associated cognitive decline increased its level [44].

Functionally, the computational tool PICRUSt predicted that tryptophan metabolism in the gut microbiota was one of the metabolic pathways significantly regulated by S-VLNs (Figure 5C). Interestingly, untargeted metabolomics analysis also highlighted the remarkable changes in this metabolic pathway (Figure 7C). Specifically, S-VLNs significantly decreased the fecal level of 4-(2-Aminophenyl)-2,4-dioxobutanoic acid and KYNA (Figure 7A,B). Tryptophan metabolism, mainly through kynurenine pathway, plays a key role in cognitive function and has been implicated in AD progression [45]. KYNA, one of the end products of kynurenine pathway, serves as an antagonist of the N-methyl-D-aspartate receptor and alpha-7-nicotinic acetylcholine receptor in the brain and an agonist of the G–protein–coupled receptor GPR35 in the enteric nervous system [25,46]. KYNA at its physiological concentration is generally neuroprotective [25], but an abnormal increase in KYNA levels in the brain has been observed in aged individuals [47], as well as in patients with neurodegenerative diseases such as AD, Parkinson’s disease, and Huntington’s disease [48]. A reduction in KYNA levels was associated with improved cognitive performance under dementia conditions [48]. Therefore, in our study, an S-VLN-mediated decrease in KYNA levels may contribute to cognitive improvement.

Notably, our integrative analysis suggested that KYNA positively correlated with five genera but negatively correlated with twelve genera (Figure 8). Two out of these seventeen genera were reported to correlate with KYNA production. In a mouse model of ventricular remodeling (a heart disease), *Enterorhabdus* was found to inversely correlate with the KYNA levels, and supplementation of probiotics containing *Enterorhabdus* significantly reduced the KYNA levels [49]. In stress-induced adolescent depressed mice, the oral administration of *Roseburia* was shown to suppress indoleamine 2,3–dioxygenase 1 (a key enzyme in the kynurenine pathway) and thus decrease the level of kynurenine, which is the direct upstream substrate of KYNA [50]. Therefore, it is tempting to speculate that orally administered S-VLNs interact with the gut microbiota, regulate the abundance and functions of specific genera (such as *Enterorhabdus* and *Roseburia*), and thus reduce the KYNA levels, which may eventually contribute to memory retention in aged mice.

Currently, no effective medications are available to manage normal age-associated cognitive decline or the pre-dementia condition MCI. Recently, microbiota-based therapies, include supplementation of probiotics/prebiotics and FMT, have been shown to improve cognitive function in mouse models of neurodegenerative disease and clinical trials with patients with AD or MCI [11,51]. For example, 16-week consumption of *Bifidobacterium breve* A1 led to a significant improvement in cognitive function in patients with MCI [52]. FMT from healthy wild–type mice mitigated amyloid β plaque formation and cognitive dysfunction in AD-like pathology with amyloid and neurofibrillary tangles (ADLP^APT^) mice [53]. Despite these promising results, the optimal type, dose, and duration of probiotics, prebiotics, or FMT treatments need to be determined in the future clinical studies. In addition, probiotic supplementation is often challenged by transient colonization and a risk of undesirable strain contamination, and prebiotics can cause gastrointestinal discomfort such as bloating, cramping, and diarrhea [54]. FMT has been associated with potential adverse effects such as pathogen transmission-related infections, gastrointestinal symptoms, or possible chronic disease transmission [55].

In recent years, dietary interventions, including supplementation of specific nutrients (omega-3 fatty acids, B vitamins, antioxidants, or their combinations) and specific diets (ketogenic diet or Mediterranean-dash intervention for neurodegenerative delay [MIND] diet), have been shown to improve cognitive function [13]. In a clinical trial with elderly patients with MCI, a combination of omega-3 and omega-6 fatty acids and antioxidant vitamins ameliorated cognitive decline [56]. Another study showed that a 6-month intake of a ketogenic drink positively correlated with cognitive improvement in patients with MCI [57]. However, some negative results of dietary interventions [13] underscore the importance of further investigation with increased sample size, extended follow-up periods, and rigorous experimental design, before they are recommended or implemented in clinical settings.

Our study revealed that S-VLNs promoted cognitive function and reshaped the gut microbiota and fecal metabolome in naturally aged mice. Unlike single–nutrient supplements, S-VLNs contain a variety of biomolecules (proteins, lipids, and nucleic acids), which could potentially target different microbial populations and metabolic and signaling pathways. Compared with specific diet approaches, the oral intake of S-VLNs is user-friendly and does not require a significant change in dietary habits, which may lead to improved adherence. From the gut microbiota perspective, S-VLNs gradually and broadly reshape the gut microbial population and functions, which solves the transient colonization issues associated with probiotic approaches and reduces the risk of FMT-related transmission in pathogen/chronic diseases. Therefore, S-VLNs represent a novel and superior potential dietary approach with the ability of modulating gut microbiota to combat cognitive dysfunction.

## 5. Conclusions

In conclusion, we found that oral intake of S-VLNs improved cognitive function and reshaped gut microbiota and fecal metabolome in aged mice. In the future, the impact of S-VLNs on cognitive function and the gut microbiota in aged humans warrants further investigation to establish their translational potential. It would also be interesting to assess the effects of S-VLNs in MCI, AD, or other neurodegenerative diseases. In our study, the causality between microbial changes and cognitive improvement or between microbial genera and metabolite level changes remain undetermined. Additional studies are needed to experimentally assess how S-VLNs modulate the growth, functions, and metabolite production in specific genera, how such changes impact cognitive function, and how specific genera regulate the level of certain metabolites.

## Data Availability

Data supporting reported results are included in the main figures and Supplementary Materials.

## Supplementary Materials

The following supporting information can be downloaded at: https://www.mdpi.com/article/10.3390/nu17172902/s1, Figure S1: S-VLNs showed no impact on the tissue structure and cellular morphology of the major metabolic tissues; Figure S2: The S-VLN treatment significantly changed the gut microbiota composition at the phylum level; Figure S3: The RDA diagram showed the strength and directions of correlations between bacterial genera and fecal metabolites; Table S1: Levels of 33 genera were significantly affected by S-VLN treatment; Table S2: Metabolites from positive and negative modes; Table S3: 66 fecal metabolites were frequently found to highly correlate with 18 bacterial genera.

## Abbreviations

The following abbreviations are used in this manuscript:

AD	Alzheimer’s Disease
ASVs	Amplicon Sequence Variants
dd-MS2	data dependent- Mass Spectrometry2
ESI	ElectroSpray Ionization
FMT	Fecal microbiota transplantation
GI	Gastrointestinal
HCA	Hierarchical Cluster Analysis
H&E	Hematoxylin and Eosin
ipGTT	intraperitoneal Glucose Tolerance Test
KEGG	Kyoto Encyclopedia of Genes and Genomes
KYNA	Kynurenic Acid
MCI	Mild Cognitive Impairment
MIND	Mediterranean-dash intervention for neurodegenerative delay
MS	Mass Spectrometry
NMDS	Non-MetricMulti-Dimensional Scaling
OPLS-DA	Orthogonal Partial Least Squares-Discriminant Analysis
PBS	Phosphate-Buffered Saline
PICRUSt	Phylogenetic Investigation of Communities by Reconstruction of Unobserved States
RDA	Redundancy Analysis
RER	Respiratory Exchange Ratio
rRNA	ribosomal RNA
S-VLNs	Shiitake Mushroom-Derived Vesicle-Like Nanoparticles
TEM	Transmission Electron Microscopy
TLC	Thin-Layer Chromatography
UPLC	Ultra-Performance Liquid Chromatography
VLNs	Vesicle-Like Nanoparticles
VIP	Variable Importance in Projection
