# Supplementary material for: Shiitake Mushroom-Derived Vesicle-like Nanoparticles Improve Cognitive Function and Reshape Gut Microbiota and Fecal Metabolome in Aged Mice
Source: Nutrients. 2025 Sep 8;17(17):2902. doi: 10.3390/nu17172902 (PMC12430123; doi:10.3390/nu17172902)
Supplement: Supplementary file 1 [file nutrients-17-02902-s001.zip › nutrients-3832144-supplementary/supplementary files/2-supple figures-r2.pdf]

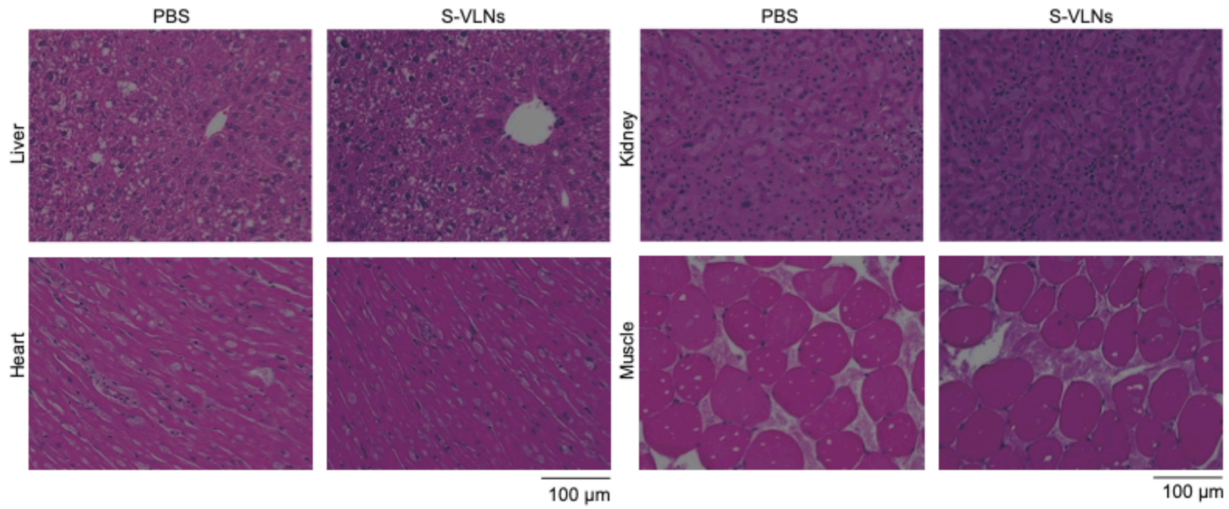

Supplementary Figure 1. S-VLNs showed no impact on the tissue structure and cellular morphology of the major metabolic tissues. The same mice from Figure 2 were euthanized when they reached 22 months of age. The major metabolic tissues including the liver, heart, kidneys, and gastrocnemius muscle were fixed in formalin, and tissue sections were subjected to H&E staining.

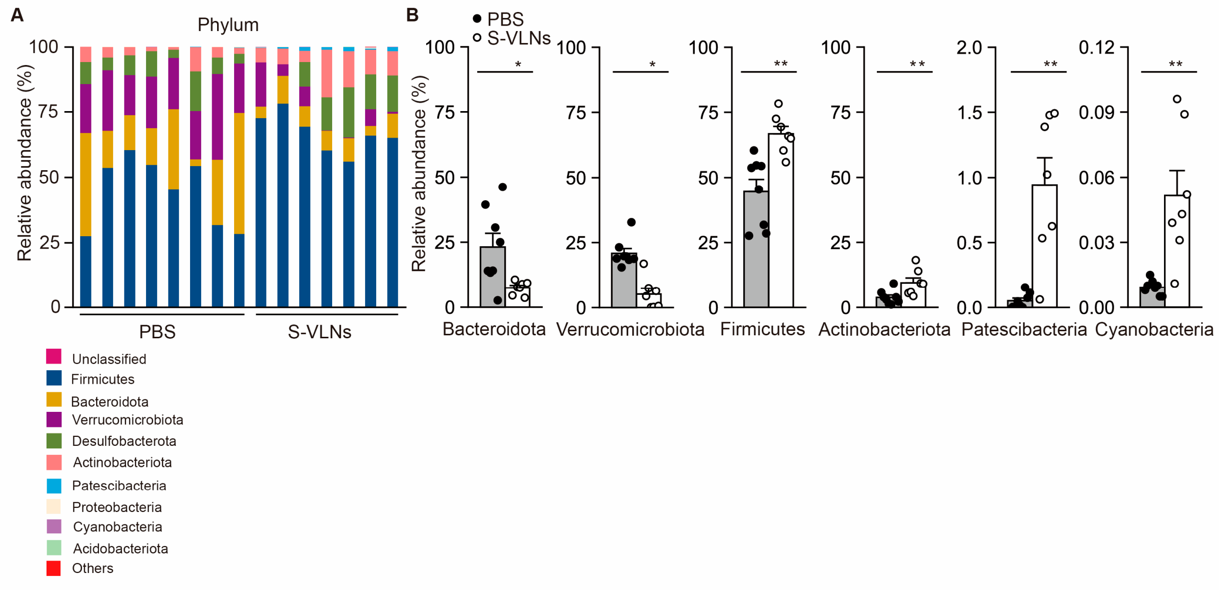

Supplementary Figure 2. The S-VLN treatment significantly changed the gut microbiota composition at the phylum level. The fecal samples of the same mice from Figure 2 were subjected to 16S rRNA analysis, followed by taxonomic composition analysis. n=7–8 mice/group. (**A**) Histogram of taxonomy distribution at the phylum classification level. Each color represents a taxonomy, and the length of the colored blocks indicates the relative abundance of the taxonomy. Only the top nine most abundant phyla were displayed in the histogram. (**B**) S-VLNs decreased Bacteroidota and Verrucomicrobiota, but increased the abundance of Firmicutes, Actinobacteriota, Patescibacteria, and Cyanobacteria. In the bar graphs, each dot represents one mouse. Data are presented as mean ± SEM. * $p < 0.05$ and ** $p < 0.01$ compared with the PBS control group (bar with black dots).

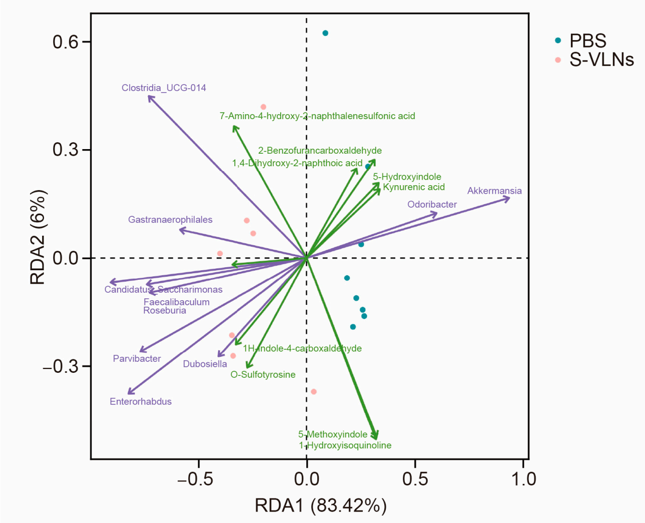

Supplementary Figure 3. The RDA diagram showed the strength and directions of correlations between bacterial genera and fecal metabolites. The top 10 metabolites and the top 10 microbial genera with the highest |r| values and $p < 0.05$ were selected for this diagram. The green arrow vectors represent metabolite variables, and the purple arrow vectors represent microbial genus variables. Acute angle between two variables indicates a positive correlation and an obtuse angle between two variables indicates a negative correlation.
